# Supplementary material for: Hyperspectral image analysis for classification of multiple infections in wheat
Source: Plant Methods. 2025 Nov 7;21:144. doi: 10.1186/s13007-025-01461-x (PMC12595906; doi:10.1186/s13007-025-01461-x)
Supplement: Supplementary file 1 — Supplementary Material 1. [file 13007_2025_1461_MOESM1_ESM.docx]

Hyperspectral image analysis for classification of multiple infections in wheat: Supplementary Materials

Manon Chossegros^1,2^, Amelia Hubbard^3^, Megan Burt^3^, Richard J. Harrison^3,4^, Charlotte F. Nellist^3^ and Nastasiya F. Grinberg^3,5^

**a**
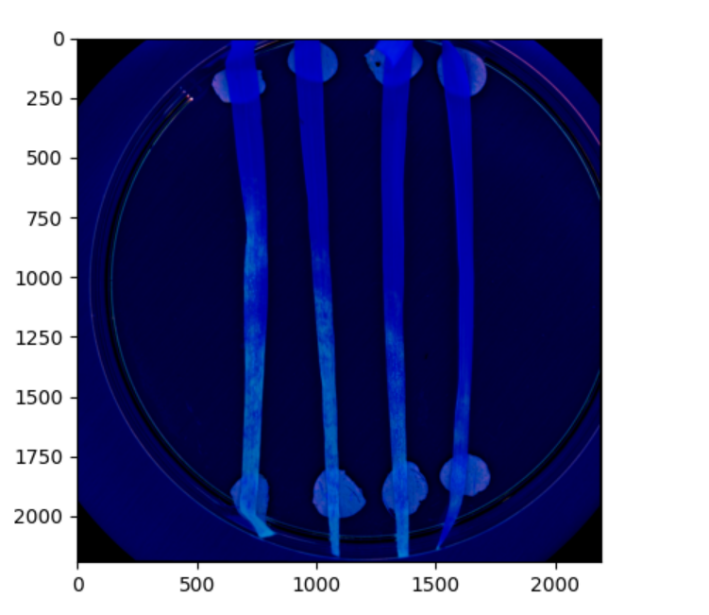
 **b**
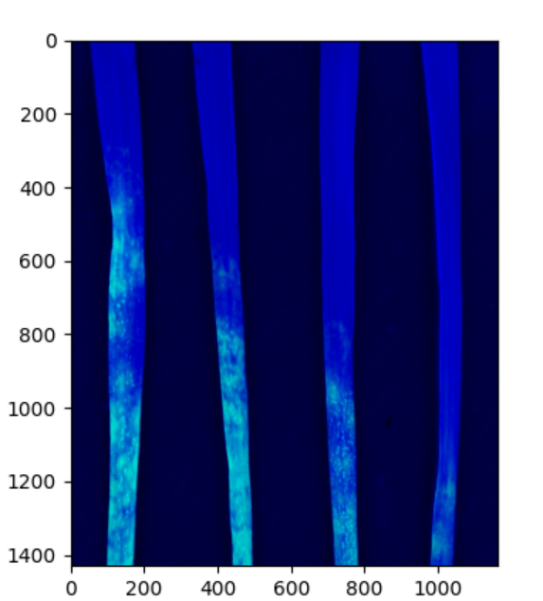


**Supplementary Figure 1** Cropping and preprocessing of the image: a) the original image, b) image after noise reduction and cropping.

**a**
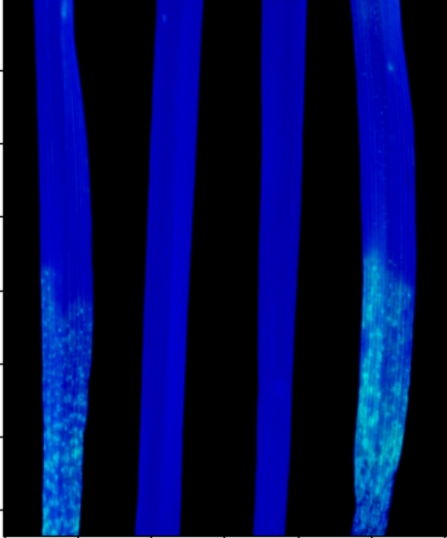
 **b**
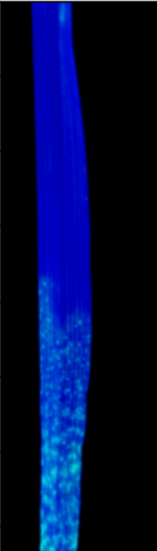

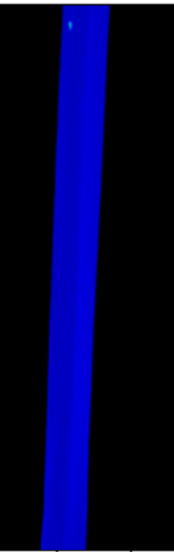

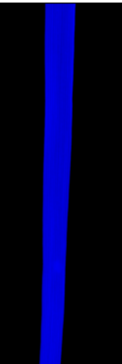

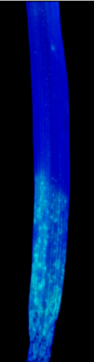


**Supplementary Figure 2** Final leaf separation: a) initial image b) separation into individual leaves -- input samples.

**a**
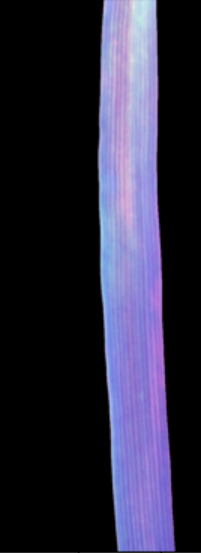
 **b**
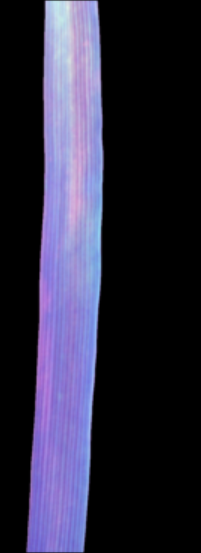
 **c**
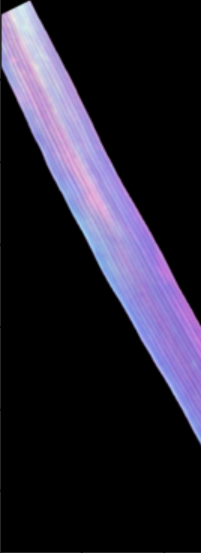


**Supplementary Figure 3** Data augmentation: a) original image, b) vertical flipping, c) random rotation.

**Supplementary Table 1** Adjusted (Bonferroni correction) p-values for the pairwise t-tests between average spectra for each disease, or pair of diseases, and uninoculated leaves, for each of the 19 wavelengths. p-values corresponding to the four largest tests statistics for each disease class are highlighted in bold.

| **Wavelength** | **Mildew** | | **Yellow rust** | **Septoria** | **YR + septoria** | **YR + mildew** |
| --- | --- | --- | --- | --- | --- | --- |
| **375** | 3.14 x ${10}^{-4}$ | 2.14 x ${10}^{-30}$ | | 6.74 x ${10}^{-9}$ | 3.05 x ${10}^{-34}$ | 7.70 x ${10}^{-23}$ |
| **405** | 4.45 x ${10}^{-4}$ | 1.78 x ${10}^{-104}$ | | 2.96 x ${10}^{-19}$ | 2.95 x ${10}^{-11}$ | 2.11 x ${10}^{-20}$ |
| **435** | 1.22 x ${10}^{-6}$ | 7.63 x ${10}^{-109}$ | | 2.60 x ${10}^{-9}$ | 4.11 x ${10}^{-53}$ | 1.20 x ${10}^{-10}$ |
| **450** | 8.88 x ${10}^{-6}$ | 2.05 x ${10}^{-123}$ | | 9.58 x ${10}^{-130}$ | 1.46 x ${10}^{-151}$ | 7.87 x ${10}^{-23}$ |
| **470** | 6.81 x ${10}^{-2}$ | 1.26 x ${10}^{-148}$ | | 7.32 x ${10}^{-180}$ | 4.80 x ${10}^{-197}$ | 1.25 x ${10}^{-38}$ |
| **505** | 1 | 1.47 x ${10}^{-191}$ | | 2.38 x ${10}^{-204}$ | 7.61 x ${10}^{-223}$ | 2.79 x ${10}^{-62}$ |
| **525** | 4.73 x ${10}^{-5}$ | 4.11 x ${10}^{-160}$ | | 3.90 x ${10}^{-52}$ | 1.41 x ${10}^{-62}$ | 2.26 x ${10}^{-8}$ |
| **570** | 9.94 x ${10}^{-4}$ | 2.43 x ${10}^{-177}$ | | 1.26 x ${10}^{-67}$ | 2.36 x ${10}^{-91}$ | 1.34 x ${10}^{-24}$ |
| **590** | 2.83 x ${10}^{-6}$ | 3.87 x ${10}^{-279}$ | | 4.19 x ${10}^{-113}$ | 1.67 x ${10}^{-143}$ | 6.21 x ${10}^{-30}$ |
| **630** | 5.84 x ${10}^{-5}$ | 0.00 | | 2.52 x ${10}^{-244}$ | 8.58 x ${10}^{-260}$ | 5.92 x ${10}^{-48}$ |
| **645** | 1.69 x ${10}^{-4}$ | **0.00** | | **0.00** | **0.00** | 2.04 x ${10}^{-66}$ |
| **660** | 6.31 x ${10}^{-3}$ | **0.00** | | **0.00** | **0.00** | **4.67 x** $\boldsymbol{10}^{\boldsymbol{-101}}$ |
| **700** | 5.33 x ${10}^{-4}$ | **0.00** | | **0.00** | **0.00** | **2.85 x** $\boldsymbol{10}^{\boldsymbol{-97}}$ |
| **780** | 1.38 x ${10}^{-3}$ | **0.00** | | **0.00** | **0.00** | 1.44 x ${10}^{-85}$ |
| **850** | 2.21 x ${10}^{-12}$ | 3.75 x ${10}^{-39}$ | | 2.26 x ${10}^{-87}$ | 9.42 x ${10}^{-75}$ | 1.27 x ${10}^{-39}$ |
| **870** | **7.56 x** $\mathbf{10}^{\mathbf{-14}}$ | 4.67 x ${10}^{-53}$ | | 8.46 x ${10}^{-34}$ | 5.24 x ${10}^{-88}$ | 1.37 x ${10}^{-69}$ |
| **890** | **2.54 x** $\mathbf{10}^{\mathbf{-15}}$ | 6.30 x ${10}^{-59}$ | | 9.02 x ${10}^{-102}$ | 2.67 x ${10}^{-115}$ | 1.69 x ${10}^{-96}$ |
| **940** | **1.31 x** $\mathbf{10}^{\mathbf{-16}}$ | 1.39 x ${10}^{-91}$ | | 1.43 x ${10}^{-39}$ | 2.21 x ${10}^{-198}$ | **1.11 x** $\boldsymbol{10}^{\boldsymbol{-155}}$ |
| **970** | **3.87 x** $\mathbf{10}^{\mathbf{-17}}$ | 4.61 x ${10}^{-143}$ | | 8.44 x ${10}^{-42}$ | 4.95 x ${10}^{-245}$ | **6.24 x** $\boldsymbol{10}^{\boldsymbol{-185}}$ |

**Supplementary Table 2** Adjusted (Bonferroni correction) p-values for the pairwise t-tests between average spectra for the individual diseases and combinations of diseases containing them, for each of the 19 wavelengths. p-values significant at 5% are highlighted in bold.

| **Wavelength** | **Mildew vs  YR + mildew** | **Yellow rust vs YR + mildew** | **Septoria vs  YR + septoria** | **Yellow rust vs YR + septoria** |
| --- | --- | --- | --- | --- |
| **375** | **6.20 x** $\boldsymbol{10}^{\boldsymbol{-4}}$ | 1 | **1.70 x** $\boldsymbol{10}^{\boldsymbol{-11}}$ | 1 |
| **405** | 2.54 x ${10}^{-1}$ | **106 x** $\boldsymbol{10}^{\boldsymbol{-6}}$ | **5.46 x** $\boldsymbol{10}^{\boldsymbol{-4}}$ | **6.58 x** $\boldsymbol{10}^{\boldsymbol{-5}}$ |
| **435** | 1.09 x ${10}^{-1}$ | **4.19 x** $\boldsymbol{10}^{\boldsymbol{-7}}$ | **8.39 x** $\boldsymbol{10}^{\boldsymbol{-2}}$ | 6.55 x ${10}^{-1}$ |
| **450** | 5.46 x ${10}^{-1}$ | **2.96 x** $\boldsymbol{10}^{\boldsymbol{-5}}$ | 1 | 1 |
| **470** | 1 | **1.66 x** $\boldsymbol{10}^{\boldsymbol{-4}}$ | 1 | 3.64 x ${10}^{-1}$ |
| **505** | 1 | **6.95 x** $\boldsymbol{10}^{\boldsymbol{-5}}$ | 1 | 9.83 x ${10}^{-1}$ |
| **525** | 1 | **2.71 x** $\boldsymbol{10}^{\boldsymbol{-14}}$ | 1 | **4.38 x** $\boldsymbol{10}^{\boldsymbol{-11}}$ |
| **570** | 1 | **4.70 x** $\boldsymbol{10}^{\boldsymbol{-13}}$ | 1 | **8.18 x** $\boldsymbol{10}^{\boldsymbol{-10}}$ |
| **590** | 1.19 x ${10}^{-1}$ | **5.49 x** $\boldsymbol{10}^{\boldsymbol{-19}}$ | 1 | **5.68 x** $\boldsymbol{10}^{\boldsymbol{-22}}$ |
| **630** | 1.02 x ${10}^{-1}$ | **8.21 x** $\boldsymbol{10}^{\boldsymbol{-18}}$ | 1 | **9.22 x** $\boldsymbol{10}^{\boldsymbol{-15}}$ |
| **645** | **4.79 x** $\boldsymbol{10}^{\boldsymbol{-2}}$ | **3.21 x** $\boldsymbol{10}^{\boldsymbol{-16}}$ | **1.90 x** $\boldsymbol{10}^{\boldsymbol{-2}}$ | **1.48 x** $\boldsymbol{10}^{\boldsymbol{-5}}$ |
| **660** | **3.64 x** $\boldsymbol{10}^{\boldsymbol{-2}}$ | **5.33 x** $\boldsymbol{10}^{\boldsymbol{-15}}$ | **6.80 x** $\boldsymbol{10}^{\boldsymbol{-4}}$ | 2.52 x ${10}^{-1}$ |
| **700** | **1.05 x** $\boldsymbol{10}^{\boldsymbol{-2}}$ | **1.77 x** $\boldsymbol{10}^{\boldsymbol{-14}}$ | **9.92 x** $\boldsymbol{10}^{\boldsymbol{-6}}$ | 1 |
| **780** | **7.58 x** $\boldsymbol{10}^{\boldsymbol{-2}}$ | **5.04 x** $\boldsymbol{10}^{\boldsymbol{-15}}$ | **1.27 x** $\boldsymbol{10}^{\boldsymbol{-3}}$ | 1 |
| **850** | **2.16 x** $\boldsymbol{10}^{\boldsymbol{-2}}$ | 1 | 1 | 1 |
| **870** | **1.72 x** $\boldsymbol{10}^{\boldsymbol{-2}}$ | 3.38 x ${10}^{-1}$ | 1.49 x ${10}^{-1}$ | 1 |
| **890** | **1.24 x** $\boldsymbol{10}^{\boldsymbol{-2}}$ | **2.35 x** $\boldsymbol{10}^{\boldsymbol{-4}}$ | 1 | 1 |
| **940** | **9.30 x** $\boldsymbol{10}^{\boldsymbol{-2}}$ | **1.37 x** $\boldsymbol{10}^{\boldsymbol{-6}}$ | **1.40 x** $\boldsymbol{10}^{\boldsymbol{-11}}$ | **4.05 x** $\boldsymbol{10}^{\boldsymbol{-3}}$ |
| **970** | 2.93 x ${10}^{-1}$ | **2.36 x** $\boldsymbol{10}^{\boldsymbol{-6}}$ | **2.34 x** $\boldsymbol{10}^{\boldsymbol{-17}}$ | **1.47 x** $\boldsymbol{10}^{\boldsymbol{-2}}$ |
